# Is Osteogenesis Imperfecta Associated with Cardiovascular Abnormalities? A Systematic Review of the Literature

**DOI:** 10.1007/s00223-023-01171-3

**Published:** 2024-01-19

**Authors:** Sara J. E. Verdonk, Silvia Storoni, Dimitra Micha, Joost G. van den Aardweg, Paolo Versacci, Luca Celli, Ralph de Vries, Lidiia Zhytnik, Otto Kamp, Marianna Bugiani, Elisabeth M. W. Eekhoff

**Affiliations:** 1 Department of Endocrinology and Metabolism, Amsterdam UMC Location Vrije Universiteit, De Boelelaan 1117, 1081 HV Amsterdam, The Netherlands; 2 Rare Bone Disease Center, Amsterdam, The Netherlands; 3 Amsterdam Movement Sciences, Amsterdam, The Netherlands; 4 Department of Human Genetics, Amsterdam UMC Location Vrije Universiteit, Amsterdam, The Netherlands; 5 Amsterdam Reproduction and Development, Amsterdam, The Netherlands; 6 Department of Respiratory Medicine, Amsterdam University Medical Center, Location AMC, Amsterdam, The Netherlands; 7 Department of Maternal Infantile and Urological Sciences, Sapienza University of Rome, Rome, Italy; 8 Medical Library, Vrije Universiteit, Amsterdam, The Netherlands; 9 Department of Traumatology and Orthopeadics, University of Tartu, Tartu, Estonia; 10 Department of Cardiology, Amsterdam UMC Location Vrije Universiteit, Amsterdam, The Netherlands; 11 Department of Pathology, Amsterdam UMC Location AMC, Amsterdam, The Netherlands

**Keywords:** Osteogenesis imperfecta, Collagen type I, Cardiovascular disease, Aortic root dilatation

## Abstract

Osteogenesis imperfecta (OI) is a rare genetic disorder caused by abnormal collagen type I production. While OI is primarily characterized by bone fragility and deformities, patients also have extraskeletal manifestations, including an increased risk of cardiovascular disease. This review provides a comprehensive overview of the literature on cardiovascular diseases in OI patients in order to raise awareness of this understudied clinical aspect of OI and support clinical guidelines. In accordance with the PRISMA guidelines, a systematic literature search in PubMed, Embase, Web of Science and Scopus was conducted that included articles from the inception of these databases to April 2023. Valvular disease, heart failure, atrial fibrillation, and hypertension appear to be more prevalent in OI than in control individuals. Moreover, a larger aortic root was observed in OI compared to controls. Various cardiovascular diseases appear to be more prevalent in OI than in controls. These cardiovascular abnormalities are observed in all types of OI and at all ages, including young children. As there are insufficient longitudinal studies, it is unknown whether these abnormalities are progressive in nature in OI patients. Based on these findings, we would recommend referring individuals with OI to a cardiologist with a low-threshold.

**Supplementary Information:**

The online version contains supplementary material available at 10.1007/s00223-023-01171-3.

## Introduction

Osteogenesis imperfecta (OI) is a rare genetic disorder characterized by brittle bones that are prone to fractures. The prevalence is 6–7 per 100,000 individuals [1]. OI has high genetic variability [2]. In approximately 85% of the cases, it is caused by monogenic variants in *COL1A1* or *COL1A2*, the genes encoding collagen type I [3], a protein that confers strength and structure to bones and other tissues. In about 15% of cases, OI is attributed to pathogenic variants in genes encoding proteins which are crucial for processes regulating the biosynthetic pathways of collagen type I, including its posttranslational modification and folding. As such, these processes are essential for the structural integrity and proper function of collagen type I. There are several types of OI, classified by the severity of skeletal symptoms [4]. The severity spectrum ranges from perinatal lethal OI to individuals with severe skeletal deformities to nearly asymptomatic individuals with a mild predisposition to fractures. While OI is characterized by bone fragility and bone deformities, patients also have extraskeletal manifestations, among which are the better known blue sclerae, dentinogenesis imperfecta, hearing difficulties, and pulmonary dysfunction.

Collagen type I is the most abundant protein in the human body. It provides tensile strength and support to various structures in the body, including bones, tendons, ligaments, and the skin [5]. It is also found in the cardiovascular system, namely the myocardium, the chordae tendineae, the valvular lamina fibrosa, and the vascular adventitia [6–11]. Individuals with OI are reported to have an increased risk of cardiovascular disease compared to a reference population [12]. Similarly to the general population, cardiovascular disease is a common cause of mortality in individuals with OI [13].

A different connective tissue disorder, namely Ehlers–Danlos syndrome (EDS) distinguishes multiple subtypes, including vascular EDS and cardiac-valvular EDS [14]. These types are most often caused by pathogenic variants in collagen type III, however both subtypes can also be a result of pathogenic variants in collagen type I. Patients with these forms of EDS may experience valvular heart dysfunction, atrial rupture, and may be more susceptible to aneurysms or rupture [14, 15]. OI mouse model studies have shown that collagen type I distortion can result in cardiovascular abnormalities such as lower breaking strength of large vessel walls, rupture of blood vessels, valvular abnormalities and myocardial dysfunction [9, 10, 16–20]. This indicates that abnormal collagen type I production could possibly lead to complications such as valvular heart disease and heart failure and underlie decreased blood vessels rigidity, by which aortic dilatation and aneurysm formation can be promoted.

The last review regarding cardiovascular disease in OI was published in 2015 [21]. Since then, more studies, especially echocardiographic studies in children, have been performed [22–26]. Although cardiovascular disease appears to be more prevalent in individuals with OI compared to control individuals [12, 21], there are currently no clinical guidelines addressing whether individuals with OI should be screened for cardiac or vascular abnormalities. This is due to a lack of information about the effect of altered collagen type 1 on the cardiovascular system in OI and its related cardiovascular pathology. Our review aims to provide a comprehensive overview of the literature to date reporting on cardiovascular diseases in people with OI. In this way, we aim to promote awareness about this understudied clinical aspect of OI in critical support of clinical guidelines.

## Methods

### Literature Search

This review was conducted according to the Preferred Reporting Items for Systematic Reviews and Meta-Analyses (PRISMA) guidelines [27]. To identify all relevant publications, we performed systematic searches in the bibliographic databases PubMed, Embase.com, Web of Science (Core Collection) and Scopus from inception to April 11, 2023, with support of a medical information specialist (RV).

The following search terms were used (including synonyms and closely related words) as index terms or free-text words: “Osteogenesis Imperfecta”, “*COL1A1*”, “*COL1A2*”, “Cardiovascular diseases”, “Cardiovascular system”. The initial search was performed without date or language restrictions. The complete search strategy for every database can be found in the Supplementary material.

### Selection Process

After deduplication, a total of 4249 papers were identified. Two reviewers (SV and SS) independently screened titles and abstracts of all potentially relevant publications for eligibility. Differences in judgement were resolved through a consensus procedure.

### Inclusion and Exclusion Criteria

The following inclusion criteria were used: (1) studies containing patients with osteogenesis imperfecta; (2) studies giving an adequate description of the cardiovascular abnormality and/or the cardiovascular examination performed; (3) studies published in English; (4) full text availability; and (5) case–control studies, cohort studies, and case series of at least 10 patients. The following exclusion criteria were used: (1) reviews with only previously described OI cases; (2) studies focusing on animal models.

### Quality Assessment

The full text of the selected articles was obtained for further review. Two reviewers (SV and SS) independently evaluated the methodological quality of the full text papers using the Study Quality Assessment Tool created by NHLBI [28].

## Results

### Search Results

**Fig. 1:**
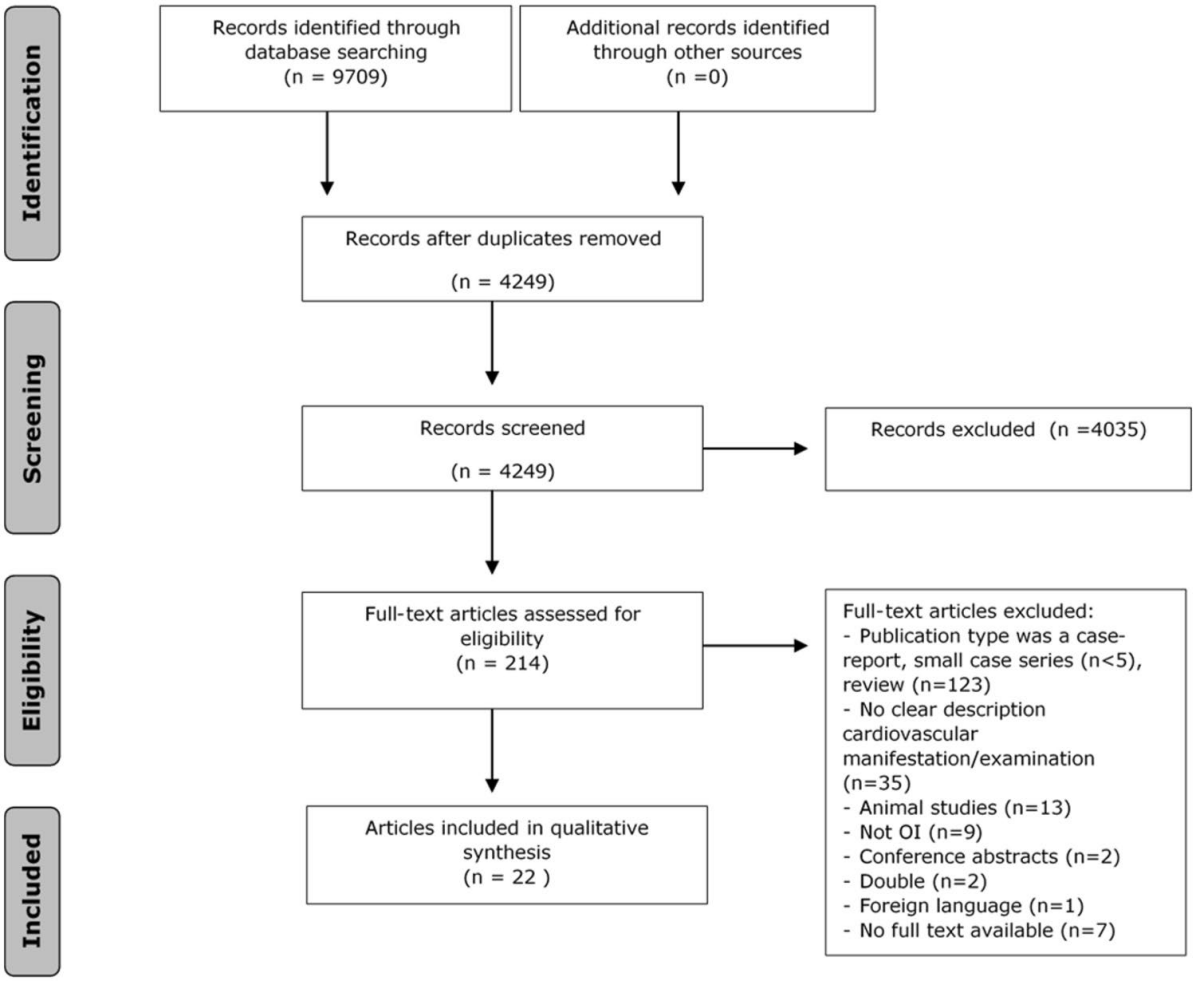
Flow chart of the study selection process

The literature search generated a total of 9709 references: 2163 in PubMed, 2541 in Embase.com, 1871 in Web of Science and 3134 in Scopus. After removing duplicates of references that were selected from more than one database, 4249 references remained. The flow chart of the search and selection process is presented in Fig. 1. Of the total 4249 articles that were identified, 214 were included for full text analysis. In total 22 articles were included. Twelve case–control studies [12, 22–26, 29–34] and ten large case series (*n* > 10)/cohort studies [10, 35–43] were identified. A quality assessment (NHLBI) of the included articles was performed and showed that 10% of the studies were classified as poor, 37% as fair and 53% as good.

### Cohort and Case Series/Cohort Studies

We classified the various findings of the cohort and case–control studies according to distinctive cardiovascular diseases, which we will specify in the next paragraphs. As the case–control studies by Radunovic ’11,’12,’15 used the same cohort, these studies have been contemplated as one study [29, 30, 34]. The demographics and results of the case–control studies can be found in Table 1 and 2. Demographics and findings of case series/cohort studies are presented in Table 3.

**Table 1:** Demographics case–control studies and presence of valvular abnormalities

First author (year)	Type of study	Country recruited patients		*n*	Mean age in years ± SD	Female, *n (*%)	BSA, m^2^ ± SD	OI type, *n*				Valvulopathies, *n* (%)				
								I	III	IV	Other	Total	AR	MR	PR	TR
Folkestad (2016) [12]	Retrospective register-based cohort study	Denmark	Cases	687	33.6^a^	379 (55%)	NA	NA	NA	NA	NA	–	6 (0.9%)*	11 (1.6%)*	NA	NA
			Controls	3435	33.5^a^	1895 (55%)	NA					–	6 (0.02%)	8 (0.2%)	NA	NA
*Adults*																
Hernández Jiménez (2018) [31]	Study by echocardiography	Spain	Cases	82	31.3 ± 15.41	47 (57.3%)	1.61 ± 0.23*	NA	NA	NA	NA	17 (20.7%)	14 (17.1%)	3 (3.7%)	–	–
			Controls	60	35.1 ± 17.22	31 (51.7%)	1.80 ± 0.25					15 (25%)	14 (23.3%)	1 (1.7%)	–	–
Kalath (1987) [32]	Study by echocardiography	US	Cases	31^b^	NA	NA	NA	24	–	7	–	NA	NA	NA	NA	NA
			Controls	50	NA	NA	NA					NA	NA	NA	NA	NA
Migliaccio (2009) [33]	Study by echocardiography	Italy	Cases	40	40	21 (52.5%)	NA	35	3	2	–	38 (95%)*	16 (40%)	38 (95%)	24 (60%)	
			Controls	40	41	20 (50%)	NA					1 (2.5%)	0	1 (2.5%)	0	
Radunovic (2011, 2012, 2015) [29, 30, 34]	Study by echocardiography	Norway	Cases	99	43.9 ± 12.3	57 (57.6%)	1.65 ± 0.31*	77	10	11	1	–	20 (20.2%)	64 (94.6%)	36 (36.4%)	91 (91.9%)
			Controls	52	43.7 ± 13.9	30 (57.7%)	1.86 ± 0.20					–	0	32 (61.5%)	24 (46.2%)	49 (94.2%)
*Children*																
Al-Senaidi (2015) [22]	Study by echocardiography	Oman	Cases	8	7.3 ± 4.3	5 (62.5%)	0.60 ± 0.28*	–	7	1	–	0	–	–	–	–
			Controls	24	6.9 ± 2.5	13 (54.2%)	0.89 ± 0.19					0	–	–	–	–
Karamifar (2013) [23]	Study by echocardiography	Iran	Cases	24	4.9 ± 3.6	NA	0.54 ± 0.17*	NA	NA	NA	NA	–	2 (8.3%)	–	1 (4.2%)	5 (20.8%)
			Controls	24	4.8 ± 3.5	NA	0.68 ± 0.25					–	NA	NA	NA	NA
Pinheiro (2020) [24]	Study by echocardiography	Brazil	Cases	54	11.16 ± 4.36*	26 (48.1%)	1.11 ± 0.38	35	18	–	1	–	–	8 (14.8%)	–	26 (48.1%)*
			Controls	54	8.20 ± 4.03	23 (42.6%)	1.09 ± 0.38					–	–	2 (3.7%)	–	8 (14.8%)
Rush (2017) [25]	Study by echocardiography	US	Cases	100	9.6 ± 4.1	55 (55%)	1.09 ± 0.47	44	54	–	2	NA	NA	NA	NA	NA
			Controls	100	9.2 ± 8.5	56 (56%)	1.08 ± 0.47					NA	NA	NA	NA	NA
Zhao (2022) [26]	Study by echocardiography	China	Cases	69	9.2 ± 4.3	28 (39.2%)	1.12 ± 0.39	42	6	17	4	26 (37.7%)*	–	NA	–	NA
			Controls	42	8.7 ± 5.1	14 (33.3%)	1.09 ± 0.45					2 (4.8%)	–	NA	–	NA

*AR* aortic valve regurgitation/insufficiency, *BSA* body surface area in m^2^, *MR* mitral valve regurgitation/insufficiency, *n* number of patients, *NA* not available, *OI* osteogenesis imperfecta, *PR* pulmonary valve regurgitation/insufficiency, *SD* standard deviation, *TR* tricuspid valve regurgitation/insufficiency

^a^Mean age at end observation

^b^From nine families

*Statistically significant difference (*P* < 0.05) between cases and controls

**Table 2:**
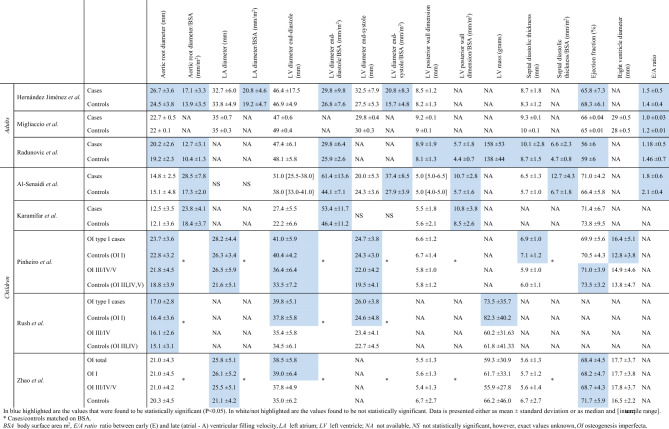
Summary of case–control studies

**Table 3:** Summary of case series/cohort studies

First author (year)	Study type	Country recruited patients	Patients *n*	Mean age ± SD, (age range)	Females *n*, (%)	OI type				Abnormal cardiac findings					
						I	III	IV	NA	Patients	Valvulopathies	Cardiac dimensions/myocardial function	Aortic root dilatation	Congenital defects	Hypertension
*Children*															
El Abd (2015) [35]	Retrospective study	Egypt	35	6.34 ± 4.85 (2 months–18 years)	17 (48.6%)	–	35	–	–	8 (22.9%)	2 (5.7.%): 1 MVP + MR + AVP 1 MVP (9 trivial TR)	Ejection fraction: mean 68,43% (59–80%) 1 patient with an ASD died during follow-up because of heart failure	1 (2.9%)	5 (14.3%): 2 ASD 2 PDA 1 VSD	NA
Vetter (1989) [40]	Prospective study	Germany	58	(1–16 years)	NA	18	25	–	15	NA	2 (3.4%): 2 MVP (OI III)	Septal and posterior wall thickness was in 95% within normal in OI type I and unclassified patients In OI type III, septal thickness and posterior left ventricular wall thickness were in 40% and 68%, respectively, above + 2SD	In OI type III, 28% of aortic diameter values were above + 2SD	4 (6.9%): 1 valvular aortic stenosis 2 ASD 1 Tetralogy of Fallot	NA
Vetter (1991) [41]	Longitudinal cohort study	Germany 117 Austria, Sweden, Italy, Turkey 10	127	Diagnosed in 1st or 2nd year of life, follow-up 10 years	NA	40	39	48	–	7 (5.5%)	NA	NA	NA	7 (5.5%): 3 ASD 2 VSD 1 aortic stenosis 1 Tetralogy of Fallot Of which 5% in OI I, 10% in OI III, 2% in OI IV	NA
Ahmad (2021)[43]	Prospective observational cross-sectional study	Saudi Arabia	23^a^	8.5 ± 4.5y (1.5–17y)	9 (39.1%)	1	16	6	–	6/19 (31.6%)	1/19 (5.3%): 1 MVP + MR (12 trivial MR or TR)	All had good cardiac function 2 patients had LV dimensions with a z-score of + 2SD	0/19	5/19 (26.3%): 1 PDA 2 PFO 2 ASD	NA
*Children and adults*															
Hortop (1986) [36]	Cohort	US	109^b^	27 years (1–75 years)	53 (48.6%)	83	16	10	–	NA	4 (3.7%): 2 AR 1 mild aortic valve stenosis 1 MVP	Left atrial dimensions were 88.2% of predicted value, left ventricular dimensions were 103.2% of predicted value	8/66 (12.1%)	NA	3 (5.7%)
Maioli (2019) [38]	Prospective study	Italy	203	NA	NA	NA	NA	NA	–	52 (25.6%), most often in adults	48 (23.6%): 21 MR 8 TR 7 AR 4 PR 3 MVP 2 MR + AR 2 MR + TR 1 MR + TR + PR	NA	NA	4 (2.0%): 1 ASD 1 Tetralogy of Fallot 1 PDA 1 LVNC	NA
Takken (2004) [39]	Intervention cohort	the Netherlands	17	13.3 ± 3.9y (8–21)	15 (88.2%)	17	–	–	–	2 (11.8%)	0 (1 trivial MR)	NA	NA	NA	NA
Thiele (2012) [10]	Longitudinal cohort	US	46	12 ± 4.2y (3–23)	27 (58.7%)	–	23	23	–	36 (78.3%)	33 (71.7%): 3 mild AR 10 mild MR 1 moderate MR 1 MVP 1 mild PR 31 mild TR	5 patients had mild left atrial enlargement	NA	3 (6.5%) 3 ASD (OI III)	NA
White (1983) [42]	Prospective study	UK	20	(13–67y)	15 (75%)	16	–	3	1	NA	2 (10.0%) 1 AR 1 MVP	1 patient had large end-systolic dimensions, this patient also had aortic valve regurgitation	6/19 (31.6%)	NA	7 (35%)
*Adults*															
Khan (2020) [37]	Prospective observational cross-sectional study	US	30	39y (19–67)	24 (80%)	9	8	12	1	10 (43.5%)	18 (60.0%) 18 MR, TR and/or PR	Ejection fraction all within normal range	0	NA	4 (16.7%) had coronary artery disease and/or hypertension

*AR* aortic valve regurgitation/insufficiency, *ASD* atrial septal defect, *AVP* aortic valve prolaps, *LVNC* left ventricular non-compaction, *MR* mitral valve regurgitation/insufficiency, *MVP* mitral valve prolapse, *n* number of patients, *NA* not available, *OI* osteogenesis imperfecta, *PDA* patent ductus arteriosus, *PR* pulmonary valve regurgitation/insufficiency, *SD* standard deviation *TR* tricuspid valve regurgitation/insufficiency, *VSD* ventricular septal defect

^a^Echocardiography was performed in 19 out of 23 children. ^b^From 66 separate families

#### Vascular Aneurysms and/or Dissections

The retrospective Danish health registry-based study by Folkestad et al*.* identified no statistically significant difference in the prevalence of vascular dissection or aneurysm diagnosis between OI and the reference population (five age-matched groups of individuals from the general population) [12]. No distinction between OI types could be made. No other case–control or case series/cohort studies investigated the presence of vascular dissections or aneurysms in OI patients. Dilatation of the aortic root is discussed separately in Sect. “Aortic Root Dilatation”.

#### Congenital Cardiovascular Defects

The literature search yielded no case–control studies on congenital cardiovascular defects. In six case series/cohort studies, OI cases with congenital cardiovascular defects were reported (Table 3). The six studies included in total 492 patients with OI of whom 28 patients (5.7%) were found to have a congenital/structural heart defect.

#### Valvular Heart Disease

While the prevalence of valve regurgitation in individuals with OI varies across different studies, there is evidence to suggest that they may have a higher chance of experiencing valvular abnormalities compared to control subjects, with mitral and aortic valve regurgitation being the most commonly reported. However, many of these regurgitations are mild or considered trivial, and only a small percentage are clinically significant. In total, eight case–control studies reported the presence or absence of valve regurgitation (Table 1). Although low in absolute numbers, the retrospective study by Folkestad et al*.* determined that OI patients in Denmark have a statistically significantly higher chance of having mitral and aortic valve regurgitation compared to five age-matched control individuals (sub-hazard ratios of 4.5 and 6.7, respectively) [12]. In addition, individuals with OI had a statistically significant higher chance of being surgically treated for mitral valve regurgitation compared to control cases and they were diagnosed with valvular regurgitation at an earlier age. Studies by Zhao et al*.*, Pinheiro et al*.* and Migliaccio et al*.* also showed that OI patients had a higher chance of experiencing valvular regurgitation compared to control subjects [24, 26, 33]. In contrast, Hernández Jiménez et al*.* did not detect a difference in the prevalence of valve regurgitations between people with OI and controls [31]. The prevalence of valve regurgitations in case–control studies using echocardiography presented a wide range from 20.7% (Hernández Jiménez et al*.*) to 95% (Migliaccio et al*.*) in adults [31, 33]. Whereas in controls, the prevalence of valve regurgitations ranged between 2.5% (Migliaccio et al*.*) and 25% (Hernández Jiménez et al*.*). In children, the prevalence of valvular abnormalities ranged from 0 (Al-Senaidi et al*.*) to 37.7% (Zhao et al.) [22, 26]. The valvular abnormalities found often included mild or trivial valve regurgitations with no hemodynamic effect. However, in one study by Migliaccio et al*.*, three mitral valve prolapses were identified, accounting for 3.8% of all valvular abnormalities in that study [33]. Additionally, moderate valve regurgitations were found by Radunovic et al*.* in 9.0% of all valvular abnormalities in their studies [29, 30, 34]. Only mild and trivial valvular abnormalities were found in the controls. In cohort studies, the prevalence of valve regurgitations varied greatly (Table 3). In studies with both children and young adults the prevalence was between 3.4 and 78% whereas in studies with only adults the prevalence was 60% [10, 35–40, 42]. Many of the reported valve regurgitations were mild or considered trivial.

#### Myocardial Dysfunction/Heart Failure

Studies indicate that individuals with OI may have an increased risk of heart failure and alterations in cardiac dimensions and function, with some evidence suggesting that they may have increased left ventricular dimensions and decreased ejection fraction (Table 2). However, results are not consistent across studies, with some reporting no significant differences in cardiac measurements between OI patients and healthy controls. A clear indicator that people with OI may have a higher risk of heart failure was demonstrated in the Danish OI population [12]. They appeared to have a higher risk of being diagnosed with heart failure compared to the reference population (sub-hazard ratio: 2.3). Additionally, the average age at diagnosis was lower in the OI population compared to the reference population (58 compared to 76 years). In eight case–control studies (Table 2), echocardiographic results were reported. Radovunic et al*.* and Hernandez et al*.* found increased left ventricle (LV) dimensions in OI patients compared to controls when adjusted for BSA [30, 31]. Although within normal range, these studies found lower ejection fraction values in OI patients compared to controls. In contrast, Migliaccio et al*.* did not find a difference in LV dimension or in ejection fraction between OI and control subjects [33]. However, Migliaccio et al*.* in addition to Radunovic et al*.* did report indications of differences in diastolic myocardial function between OI and controls shown by a lower E/A ratio (ratio between early (E) and late (atrial—A) ventricular filling velocity). This was not confirmed by Hernández Jiménez et al*.*, who showed a higher E/A ratio. In OI children, increased LV dimensions were found in some studies, but not consistently across all studies. Ejection fraction was generally found to be equal between OI cases and controls, except for in Zhao et al*.* and Pinheiro et al*.* where a lower ejection fraction was found in specific subgroups [24, 26]. When stratified based on genetic variants, Zhao et al*.* found increased LA and LV diameters with lower ejection fraction for individuals with a COL1A1 variant compared to controls. Additionally, cardiac dimensions and/or myocardial function were evaluated in seven case series/cohort studies (Table 3).

#### Aortic Root Dilatation

The available evidence reported on by nine case–control studies, suggests that there may be an association between OI and increased aortic root diameters (Table 2) [32]. However, there is some inconsistency in the findings across studies, with some studies reporting no difference between OI cases and controls. Furthermore, the prevalence of an actual aortic root dilatation remains unclear. In adults, the studies by Hernandez-Jimenez et al*.* and Randunovic et al*.* demonstrated higher aortic root diameters in OI patients compared to controls. In contrast, the study by Migliaccio et al*.* did not identify a difference between aortic root diameters of OI cases and healthy controls [29, 33]. In children, higher aortic root diameters were found in OI cases compared to controls by Al-Senaidi et al*.*, Karamifar et al*.*, Pinheiro et al., and Rush et al. [22–25]. In the study of Karamifar et al*.* 5 of 24 children with OI had Z-scores > 2. Zhao et al*.* found no difference in aortic root diameter in OI individuals compared to controls, they did however find an increased pulmonic artery root in individuals with OI compared to controls [23, 26]. In the study conducted by Kalath et al*.*, 12.9% of type I or IV OI patients had a dilated aortic root [32]. Despite the lack of a direct correlation between aortic root stiffness and aortic root diameter, the results indicated an increase in aortic root stiffness relative to the control group. According to the study of Katalth et al*.* his appears consistent with aging-related alterations and suggest that premature aging of the aortic root may occur in OI. No other studies investigated aortic root stiffness. In case series/cohort studies five studies investigated aortic root dilatation (Table 3). The prevalence varied between 0 in children (Ahmad et al*.*) and 31.6% in both children and adults (White et al.) [42, 43].

#### Hypertension

Diagnosis of hypertension or use of hypertensive drugs was more prevalent in the Danish OI population compared to a reference population according to Folkestad et al*.* (prevalence 28.1 vs 21.6%) [12]. In the case–control study by Radunovic et al*.*, 37 of 99 patients (37.4%) had hypertension, although the prevalence in their control group was not reported [29]. In the large case series of Hortop et al*.* the prevalence of hypertension was 2.8% in 109 individuals 1–74 year old whereas this was 35% in 20 individuals 13–68 year old in the study of White et al. [36, 42] (Table 3).

#### Atherosclerosis

Folkestad et al*.* found no statistical significant difference in the prevalence of diagnosis of ischemic cardiovascular disease and dyslipidemia [12]. In the study by Radunovic et al*.*, two of 99 patients (2.0%) were reported to have ischemic cardiovascular disease [29]. The prevalence in the control population is not reported. In the case series by White et al*.* 1 of 20 patients (5%) was reported to have had a stroke (age unknown) [42]. This patient also had hypertension.

#### Atrial Flutter/Fibrillation

The Danish OI population presented a higher chance of atrial fibrillation or atrial flutter diagnosis compared to the used reference population (sub-hazard ratio: 1.7) [12]. No other case–control or cohort studies reported on this topic.

## Discussion

The purpose of this article was to provide a systematic review of the existing literature describing cardiovascular abnormalities in patients diagnosed with OI. We included 22 studies with both retrospective and prospective designs. Valvular heart disease, (subclinical) myocardial dysfunction, atrial fibrillation and hypertension appear to be more common in OI compared to controls. Notably, atrial fibrillation and hypertension have been examined in only a single, yet the largest study currently available [12]. In addition, a wider aortic root was observed in OI compared to controls. Cardiovascular abnormalities are reported at all ages, including early childhood and spread across all types of OI.

Collagen type I is highly abundant in the lamina fibrosa of the heart valves and is essential for the mechanical integrity [44]. According to the studies in our review, individuals with OI appear to have a higher risk of valvular regurgitation. Most case–control and cohort studies concerned almost exclusively mild regurgitations with no hemodynamic effect [23, 24, 26, 33]. On the other hand, according to Folkestad et al., mitral valve regurgitations is more frequently treated surgically in OI patients compared to controls, indicating that valvular abnormalities are clinically significant [12]. This points to valvular heart disease being clinically relevant abnormalities in OI. It remains unclear whether the risk of valvular abnormalities differs between different OI types and/or patients with different pathogenic variants. Further research is needed to assess the clinical consequences for the patients. A study performed in a severe OI mouse model (homozygous OIM mice) suggested that aortic valves are more affected than mitral valves [20]. Furthermore, collagen fibril disorganization and decreased collagen fibril diameter was found in atrioventricular valves of fetuses with OI type II [8]. It remains unclear if one heart valve is more often affected in OI compared to other heart valves.

Based on the studies included in this review, individuals with OI are more likely to have (subclinical) LV dysfunction compared to than controls. The underlying cause of heart failure in OI patients remains poorly understood. Fibrillar collagen is the most prevalent protein of the cardiac extracellular matrix; it consists mainly of collagen type I (over 80%) and collagen type III (over 10%) [45]. Since they provide tensile strength and resistance to deformation, collagen type I fibers are especially essential for maintaining the integrity and elasticity of cardiac tissue [46, 47]. In addition, they play a crucial role in transferring mechanical forces from the extracellular matrix to the cardiomyocytes and vice versa, optimizing the pumping efficiency of the heart [48, 49]. Structural and functional problems of the heart arise from collagen metabolism imbalance, as seen in cardiac fibrosis or after myocardial infarction [50]. Therefore, collagen type I defects (abnormal or less collagen type I) may affect the myocardial structure. In homozygous OIM mice, a higher risk of ventricle rupture after myocardial infarction was noticed compared to wild type mice, suggesting that deficiency of collagen I leads to a myocardial wound-healing defect [51]. However, no further studies on this topic have been published. Furthermore, patients with OI may also be at higher risk of developing other conditions that can contribute to heart failure, such as scoliosis, chronic lung disease, and obesity [52]. These comorbidities can cause additional strain on the heart and cardiovascular system, potentially leading to heart failure.

In studies using echocardiograms, both adults and children with OI had on average a larger aortic root compared to controls. While these values were often within normal ranges, there were instances in which they exceeded normal levels, such as by more than two Z-scores in children [23]. As no longitudinal studies are currently available, it is unclear whether this should be considered potentially progressive. Collagen type I accounts for approximately 60% of the collagen found in the vessel wall. It is important for the mechanical resistance of the arterial wall [53]. In homozygous OIM mice and homozygous Col-IntΔ mice, both severe OI models with a collagen type I defect, vascular aneurysms and/or ruptures are more frequent compared to wild type mice [9, 16–18]. Vascular aneurysms and dissections are reported in OI individuals in case-reports and small case series [54]. However, they do not appear to be more prevalent compared to controls, as a low absolute number was found and no differences were identified between OI individuals and the reference population, which was only investigated in one case–control study [12]. Additionally, a few case-reports reported cerebrovascular abnormalities (aneurysms and/or dissections) [55], but this has not been studied on a larger cohort, and thus not included in the results of this review. These case-reports included reports on intracranial hemorrhage after relatively minor head-trauma in OI patients, suggesting that vascular fragility may be a contributor factor in addition to cranial bone fragility [56–59].

The risk of hypertension, atrial fibrillation and arteriosclerosis in individuals with OI has been scarcely studied. People with OI examined in a retrospective study appear to be more likely to have hypertension and atrial arrhythmias, while no statistically significant difference was found for the prevalence of diagnosis of ischemic cardiovascular disease and dyslipidemia [12]. Since hypertension is common and multifactorial, with no known association to OI or collagen type I, further research is needed to elucidate its relation to OI. An explanation for a connection between OI and arrhythmias may be the role of collagen type I as component of the extracellular matrix of the cardiac conduction system, which ensures its insulation from the rest of the cardiac tissue. In this case, collagen integrity may be required for efficient transduction of the electrical signals [60, 61].

The investigation of congenital cardiovascular abnormalities in patients with OI is limited, which could indicate that patients with OI do not appear to have a (clinically relevant) higher risk of congenital defects compared to controls. However, the mean prevalence of 5.7% in OI cohort studies and large case series is higher than the approximately 1% occurrence rate in live births in the general population [62]. Septal defects and patent ductus arteriosus are the most common cardiovascular abnormalities found in OI patients, similar to the general population. Due to the limited number of cases studied, it is unclear whether the findings are coincidental or linked to abnormal collagen formation in OI.

A limitation of this review is that as a result of studies with a small number of patients, a wide range of age groups, and differences in demographic profiles and OI types, the correlational power of the findings of some studies may be insufficient. Therefore, systematic research on large cohort and case–control studies with comprehensive clinical and molecular characterization and uniform outcome measures should be encouraged. Unfortunately, for this review, it was not possible to correlate cardiovascular problems with the type of OI, given that in most studies in adults, no discrimination between OI types was made in the analyses or the OI type was unknown. In studies involving children, attempts were made at times to distinguish between mild (OI I) and severe OI (OI IV, III, or V). However, the varying characteristics across these studies and the absence of clear, consistent differences between OI I and OI IV, III, or V) presented challenges in drawing conclusions. In future research, distinction between clinical OI types and genetic defect types can hopefully help to decipher the contribution of skeletal deformities and different genetic backgrounds to the cardiovascular OI pathology. Moreover, further research is necessary to investigate the relationship between cardiovascular abnormalities in OI and age. Pathologic examination can also be invaluable for insights in the way in which structure and function of the cardiovascular system is compromised in OI. Nonetheless, this review provides valuable insights into potential cardiovascular abnormalities in OI. Currently, there is no established policy or practice regarding screening for cardiovascular abnormalities in OI. Due to existing knowledge gaps, it would be premature to provide recommendations based on the findings of this review. We do believe vigilance is deserved, and we advocate low-threshold referral for a transthoracic echocardiogram for OI patients.

In conclusion, the existing literature suggests that individuals with OI have a higher risk of cardiovascular abnormalities, including valvular heart disease, heart failure, and a wider aortic root compared to controls. Valvular abnormalities, specifically mitral valve regurgitation, appear to be clinically relevant. Although currently investigated in only a single study, atrial fibrillation and hypertension may be potentially more frequent in individuals with OI. As no longitudinal studies exist, it is unclear whether these cardiovascular abnormalities are progressive in nature in OI. The risk of hypertension, atrial fibrillation and congenital heart disease in individuals with OI requires further study. Overall, further research is necessary to understand the precise mechanism and clinical consequences of cardiovascular abnormalities in individuals with OI.

### Supplementary Information

Below is the link to the electronic supplementary material.

Supplementary file1 (DOCX 32 KB)

## Abbreviations

BSA: Body surface area
EDS: Ehlers–Danlos syndrome
OI: Osteogenesis imperfecta
LA: Left atrium
LV: Left ventricle
