# Supplementary material for: Is Osteogenesis Imperfecta Associated with Cardiovascular Abnormalities? A Systematic Review of the Literature
Source: Calcif Tissue Int. 2024 Jan 19;114(3):210–21. doi: 10.1007/s00223-023-01171-3 (PMC10902066; doi:10.1007/s00223-023-01171-3)
Supplement: Supplementary file 1 — Supplementary file1 (DOCX 32 KB) [file 223_2023_1171_MOESM1_ESM.docx]

**Appendix A / Supplementary material**

**PubMed Session Results (11 Apr 2023)**

| Search | Query | Items found |
| --- | --- | --- |
| #3 | **#1 AND #2** | 2,163 |
| #2 | **"Cardiovascular Physiological Phenomena"[Mesh] OR "Cardiovascular Diseases"[Mesh] OR "Cardiovascular System"[Mesh] OR "Hyperlipidemias"[Mesh] OR "Cholesterol"[Mesh] OR "Cardiac Imaging Techniques"[Mesh] OR "Diagnostic Techniques, Cardiovascular"[Mesh] OR cardiovascul*[tiab] OR "cardio-vascul*"[tiab] OR cvd[tiab] OR heart[tiab] OR artery[tiab] OR arteries[tiab] OR vessel[tiab] OR vessels[tiab] OR vein[tiab] OR veins[tiab] OR venous[tiab] OR vascular*[tiab] OR capillar*[tiab] OR cardiac[tiab] OR myocard*[tiab] OR coronary[tiab] OR coronaries[tiab] OR arterial[tiab] OR atrial[tiab] OR artrial[tiab] OR "angina pectoris"[tiab] OR angiogene*[tiab] OR neovasculari*[tiab] OR vasculogene*[tiab] OR (collateral[tiab] AND circulation[tiab]) OR stenosis[tiab] OR stenoses[tiab] OR stenotic[tiab] OR atherosclero*[tiab] OR arteriosclero*[tiab] OR hypertens*[tiab] OR "hyper-tens*"[tiab] OR hypotens*[tiab] OR "hypo-tens*"[tiab] OR "blood pressure"[tiab] OR cholesterol[tiab] OR stroke*[tiab] OR hypercholesterol*[tiab] OR hyperlipid*[tiab] OR arhythm*[tiab] OR aorta[tiab] OR aneurysm*[tiab] OR dissection[tiab] OR valve*[tiab] OR "sinoatrial node"[tiab] OR "sinus node"[tiab] OR tachycardia[tiab] OR hemodynamic*[tiab] OR haemodynamic*[tiab] OR ventricular*[tiab] OR atrioventricular[tiab] OR neoangiogene*[tiab] OR "neo-angiogene*"[tiab] OR endothelium[tiab] OR "bundle of his"[tiab] OR "bundle-branch block"[tiab] OR "AV block"[tiab] OR varic*[tiab] OR "tunica intima"[tiab] OR adventitia[tiab] OR "tunica media"[tiab] OR angiograph*[tiab] OR echocardiograph*[tiab] OR electrocardiograph*[tiab] OR ecg[tiab] OR holter[tiab]** | 5,788,217 |
| #1 | **"Osteogenesis Imperfecta"[Mesh] OR "osteogenesis imperfect*"[tiab] OR (brittle*[tiab] AND bone*[tiab]) OR lobstein*[tiab] OR vrolik*[tiab] OR bruck*[tiab] OR "Fragilitas Ossium"[tiab] OR "COL1A1"[tiab] OR "COL1A2"[tiab] OR "COL1-A1"[tiab] OR "COL1-A2"[tiab] OR "COL-1A1"[tiab] OR "COL-1A2"[tiab] OR "COL1A-1"[tiab] OR "COL1A-2"[tiab] OR "col1α1"[tiab] OR "col1α2"[tiab] OR "col1-α1"[tiab] OR "col1-α2"[tiab] OR "col-1α1"[tiab] OR "col-1α2"[tiab] OR "col1α-1"[tiab] OR "col1α-2"[tiab] OR "collagen1A1"[tiab] OR "collagen1A2"[tiab] OR "collagen1 A1"[tiab] OR "collagen1 A2"[tiab] OR "collagen 1A1"[tiab] OR "collagen 1A2"[tiab] OR "collagen1α1"[tiab] OR "collagen1α2"[tiab] OR "collagen1 α1"[tiab] OR "collagen1 α2"[tiab] OR "collagen 1α1"[tiab] OR "collagen 1α2"[tiab] OR "collagen type Iα1"[tiab] OR "collagen type Iα2"[tiab] OR "COL1A2 protein, human" [Supplementary Concept] OR "Col1a2 protein, mouse" [Supplementary Concept] OR "COL1A2 protein, zebrafish" [Supplementary Concept] OR "alpha 2(I) Collagen"[tiab] OR "alpha 1(I) Collagen"[tiab] OR "alpha 2 Collagen Type I"[tiab] OR "alpha 1 Collagen Type I"[tiab] OR "alpha2(I)-Collagen"[tiab] OR "alpha1(I)-Collagen"[tiab] OR "alpha-2 Type 1 Collagen"[tiab] OR "alpha-1 Type 1 Collagen"[tiab] OR "alpha2 Type 1 Collagen"[tiab] OR "alpha1 Type 1 Collagen"[tiab] OR "Collagen alpha2(I)"[tiab] OR "Collagen alpha1(I)"[tiab] OR "Collagen Type 1 alpha-2"[tiab] OR "Collagen Type 1 alpha-1"[tiab] OR "Collagen Type 1 alpha 2"[tiab] OR "Collagen Type 1 alpha 1"[tiab] OR "collagen alpha2(I)"[tiab] OR "collagen alpha1(I)"[tiab] OR "alpha2 Type 1 Collagen"[tiab] OR "alpha1 Type 1 Collagen"[tiab]** | 14,808 |

**Embase.com Session Results (11 Apr 2023)**

| Search | Query | Items found |
| --- | --- | --- |
| #4 | **#3 NOT ('conference abstract'/it OR 'conference review'/it)** | 2,541 |
| #3 | **#1 AND #2** | 4,028 |
| #2 | **'cardiovascular function'/exp OR 'cardiovascular disease'/exp OR 'hyperlipidemia'/exp OR 'cholesterol'/exp OR 'cardiac imaging'/exp OR 'cardiovascular system examination'/exp OR cardiovascul*:ab,ti,kw OR 'cardio-vascul*':ab,ti,kw OR cvd:ab,ti,kw OR heart:ab,ti,kw OR artery:ab,ti,kw OR arteries:ab,ti,kw OR vessel:ab,ti,kw OR vessels:ab,ti,kw OR vein:ab,ti,kw OR veins:ab,ti,kw OR venous:ab,ti,kw OR vascular*:ab,ti,kw OR capillar*:ab,ti,kw OR cardiac:ab,ti,kw OR myocard*:ab,ti,kw OR coronary:ab,ti,kw OR coronaries:ab,ti,kw OR arterial:ab,ti,kw OR atrial:ab,ti,kw OR artrial:ab,ti,kw OR 'angina pectoris':ab,ti,kw OR angiogene*:ab,ti,kw OR neovasculari*:ab,ti,kw OR vasculogene*:ab,ti,kw OR (collateral:ab,ti,kw AND circulation:ab,ti,kw) OR stenosis:ab,ti,kw OR stenoses:ab,ti,kw OR stenotic:ab,ti,kw OR atherosclero*:ab,ti,kw OR arteriosclero*:ab,ti,kw OR hypertens*:ab,ti,kw OR 'hyper-tens*':ab,ti,kw OR hypotens*:ab,ti,kw OR 'hypo-tens*':ab,ti,kw OR 'blood pressure':ab,ti,kw OR cholesterol:ab,ti,kw OR stroke*:ab,ti,kw OR hypercholesterol*:ab,ti,kw OR hyperlipid*:ab,ti,kw OR arhythm*:ab,ti,kw OR aorta:ab,ti,kw OR aneurysm*:ab,ti,kw OR dissection:ab,ti,kw OR valve*:ab,ti,kw OR 'sinoatrial node':ab,ti,kw OR 'sinus node':ab,ti,kw OR tachycardia:ab,ti,kw OR hemodynamic*:ab,ti,kw OR haemodynamic*:ab,ti,kw OR ventricular*:ab,ti,kw OR atrioventricular:ab,ti,kw OR neoangiogene*:ab,ti,kw OR 'neo-angiogene*':ab,ti,kw OR endothelium:ab,ti,kw OR 'bundle of his':ab,ti,kw OR 'bundle-branch block':ab,ti,kw OR 'AV block':ab,ti,kw OR varic*:ab,ti,kw OR 'tunica intima':ab,ti,kw OR adventitia:ab,ti,kw OR 'tunica media':ab,ti,kw OR angiograph*:ab,ti,kw OR echocardiograph*:ab,ti,kw OR electrocardiograph*:ab,ti,kw OR ecg:ab,ti,kw OR holter:ab,ti,kw** | 8,465,825 |
| #1 | **'osteogenesis imperfecta'/exp OR 'col1a2 protein'/exp OR 'col1a2 protein human'/exp OR 'osteogenesis imperfect*':ab,ti,kw OR (brittle*:ab,ti,kw AND bone*:ab,ti,kw) OR lobstein*:ab,ti,kw OR vrolik*:ab,ti,kw OR bruck*:ab,ti,kw OR 'Fragilitas Ossium':ab,ti,kw OR 'COL1A1':ab,ti,kw OR 'COL1A2':ab,ti,kw OR 'COL1-A1':ab,ti,kw OR 'COL1-A2':ab,ti,kw OR 'COL-1A1':ab,ti,kw OR 'COL-1A2':ab,ti,kw OR 'COL1A-1':ab,ti,kw OR 'COL1A-2':ab,ti,kw OR 'col1α1':ab,ti,kw OR 'col1α2':ab,ti,kw OR 'col1-α1':ab,ti,kw OR 'col1-α2':ab,ti,kw OR 'col-1α1':ab,ti,kw OR 'col-1α2':ab,ti,kw OR 'col1α-1':ab,ti,kw OR 'col1α-2':ab,ti,kw OR 'collagen1A1':ab,ti,kw OR 'collagen1A2':ab,ti,kw OR 'collagen1 A1':ab,ti,kw OR 'collagen1 A2':ab,ti,kw OR 'collagen 1A1':ab,ti,kw OR 'collagen 1A2':ab,ti,kw OR 'collagen1α1':ab,ti,kw OR 'collagen1α2':ab,ti,kw OR 'collagen1 α1':ab,ti,kw OR 'collagen1 α2':ab,ti,kw OR 'collagen 1α1':ab,ti,kw OR 'collagen 1α2':ab,ti,kw OR 'collagen type Iα1':ab,ti,kw OR 'collagen type Iα2':ab,ti,kw OR 'alpha 2(I) Collagen':ab,ti,kw OR 'alpha 1(I) Collagen':ab,ti,kw OR 'alpha 2 Collagen Type I':ab,ti,kw OR 'alpha 1 Collagen Type I':ab,ti,kw OR 'alpha2(I)-Collagen':ab,ti,kw OR 'alpha1(I)-Collagen':ab,ti,kw OR 'alpha-2 Type 1 Collagen':ab,ti,kw OR 'alpha-1 Type 1 Collagen':ab,ti,kw OR 'alpha2 Type 1 Collagen':ab,ti,kw OR 'alpha1 Type 1 Collagen':ab,ti,kw OR 'Collagen alpha2(I)':ab,ti,kw OR 'Collagen alpha1(I)':ab,ti,kw OR 'Collagen Type 1 alpha-2':ab,ti,kw OR 'Collagen Type 1 alpha-1':ab,ti,kw OR 'Collagen Type 1 alpha 2':ab,ti,kw OR 'Collagen Type 1 alpha 1':ab,ti,kw OR 'collagen alpha2(I)':ab,ti,kw OR 'collagen alpha1(I)':ab,ti,kw OR 'alpha2 Type 1 Collagen':ab,ti,kw OR 'alpha1 Type 1 Collagen':ab,ti,kw** | 21,305 |

**Web of Science (Core Collection) Session Results (11 Apr 2023)**

| Search | Query | Items found |
| --- | --- | --- |
| #3 | **#1 AND #2** | 1,871 |
| #2 | **TS=("cardiovascul*" OR "cardio-vascul*" OR "cvd" OR "heart" OR "artery" OR "arteries" OR "vessel" OR "vessels" OR "vein" OR "veins" OR "venous" OR "vascular*" OR "capillar*" OR "cardiac" OR "myocard*" OR "coronary" OR "coronaries" OR "arterial" OR "atrial" OR "artrial" OR "angina pectoris" OR "angiogene*" OR "neovasculari*" OR "vasculogene*" OR ("collateral" AND "circulation") OR "stenosis" OR "stenoses" OR "stenotic" OR "atherosclero*" OR "arteriosclero*" OR "hypertens*" OR "hyper-tens*" OR "hypotens*" OR "hypo-tens*" OR "blood pressure" OR "cholesterol" OR "stroke*" OR "hypercholesterol*" OR "hyperlipid*" OR "arhythm*" OR "aorta" OR "aneurysm*" OR "dissection" OR "valve*" OR "sinoatrial node" OR "sinus node" OR "tachycardia" OR "hemodynamic*" OR "haemodynamic*" OR "ventricular*" OR "atrioventricular" OR "neoangiogene*" OR "neo-angiogene*" OR "endothelium" OR "bundle of his" OR "bundle-branch block" OR "AV block" OR "varic*" OR "tunica intima" OR "adventitia" OR "tunica media" OR "angiograph*" OR "echocardiograph*" OR "electrocardiograph*" OR "ecg" OR "holter")** | 5,852,126 |
| #1 | **TS=("osteogenesis imperfect*" OR ("brittle*" AND "bone*") OR "lobstein*" OR "vrolik*" OR "bruck*" OR "Fragilitas Ossium" OR "COL1A1" OR "COL1A2" OR "COL1-A1" OR "COL1-A2" OR "COL-1A1" OR "COL-1A2" OR "COL1A-1" OR "COL1A-2" OR "col1α1" OR "col1α2" OR "col1-α1" OR "col1-α2" OR "col-1α1" OR "col-1α2" OR "col1α-1" OR "col1α-2" OR "collagen1A1" OR "collagen1A2" OR "collagen1 A1" OR "collagen1 A2" OR "collagen 1A1" OR "collagen 1A2" OR "collagen1α1" OR "collagen1α2" OR "collagen1 α1" OR "collagen1 α2" OR "collagen 1α1" OR "collagen 1α2" OR "collagen type Iα1" OR "collagen type Iα2" OR "alpha 2(I) Collagen" OR "alpha 1(I) Collagen" OR "alpha 2 Collagen Type I" OR "alpha 1 Collagen Type I" OR "alpha2(I)-Collagen" OR "alpha1(I)-Collagen" OR "alpha-2 Type 1 Collagen" OR "alpha-1 Type 1 Collagen" OR "alpha2 Type 1 Collagen" OR "alpha1 Type 1 Collagen" OR "Collagen alpha2(I)" OR "Collagen alpha1(I)" OR "Collagen Type 1 alpha-2" OR "Collagen Type 1 alpha-1" OR "Collagen Type 1 alpha 2" OR "Collagen Type 1 alpha 1" OR "collagen alpha2(I)" OR "collagen alpha1(I)" OR "alpha2 Type 1 Collagen" OR "alpha1 Type 1 Collagen")** | 18,253 |

**Scopus Session Results (11 Apr 2023)**

| Search | Query | Items found |
| --- | --- | --- |
| #3 | **#1 AND #2** | 3,134 |
| #2 | **TITLE-ABS-KEY ("cardiovascul*" OR "cardio-vascul*" OR "cvd" OR "heart" OR "artery" OR "arteries" OR "vessel" OR "vessels" OR "vein" OR "veins" OR "venous" OR "vascular*" OR "capillar*" OR "cardiac" OR "myocard*" OR "coronary" OR "coronaries" OR "arterial" OR "atrial" OR "artrial" OR "angina pectoris" OR "angiogene*" OR "neovasculari*" OR "vasculogene*" OR ("collateral" AND "circulation") OR "stenosis" OR "stenoses" OR "stenotic" OR "atherosclero*" OR "arteriosclero*" OR "hypertens*" OR "hyper-tens*" OR "hypotens*" OR "hypo-tens*" OR "blood pressure" OR "cholesterol" OR "stroke*" OR "hypercholesterol*" OR "hyperlipid*" OR "arhythm*" OR "aorta" OR "aneurysm*" OR "dissection" OR "valve*" OR "sinoatrial node" OR "sinus node" OR "tachycardia" OR "hemodynamic*" OR "haemodynamic*" OR "ventricular*" OR "atrioventricular" OR "neoangiogene*" OR "neo-angiogene*" OR "endothelium" OR "bundle of his" OR "bundle-branch block" OR "AV block" OR "varic*" OR "tunica intima" OR "adventitia" OR "tunica media" OR "angiograph*" OR "echocardiograph*" OR "electrocardiograph*" OR "ecg" OR "holter")** | 8,014,750 |
| #1 | **TITLE-ABS-KEY ("osteogenesis imperfect*" OR ("brittle*" AND "bone*") OR "lobstein*" OR "vrolik*" OR "bruck*" OR "Fragilitas Ossium" OR "COL1A1" OR "COL1A2" OR "COL1-A1" OR "COL1-A2" OR "COL-1A1" OR "COL-1A2" OR "COL1A-1" OR "COL1A-2" OR "col1α1" OR "col1α2" OR "col1-α1" OR "col1-α2" OR "col-1α1" OR "col-1α2" OR "col1α-1" OR "col1α-2" OR "collagen1A1" OR "collagen1A2" OR "collagen1 A1" OR "collagen1 A2" OR "collagen 1A1" OR "collagen 1A2" OR "collagen1α1" OR "collagen1α2" OR "collagen1 α1" OR "collagen1 α2" OR "collagen 1α1" OR "collagen 1α2" OR "collagen type Iα1" OR "collagen type Iα2" OR "alpha 2(I) Collagen" OR "alpha 1(I) Collagen" OR "alpha 2 Collagen Type I" OR "alpha 1 Collagen Type I" OR "alpha2(I)-Collagen" OR "alpha1(I)-Collagen" OR "alpha-2 Type 1 Collagen" OR "alpha-1 Type 1 Collagen" OR "alpha2 Type 1 Collagen" OR "alpha1 Type 1 Collagen" OR "Collagen alpha2(I)" OR "Collagen alpha1(I)" OR "Collagen Type 1 alpha-2" OR "Collagen Type 1 alpha-1" OR "Collagen Type 1 alpha 2" OR "Collagen Type 1 alpha 1" OR "collagen alpha2(I)" OR "collagen alpha1(I)" OR "alpha2 Type 1 Collagen" OR "alpha1 Type 1 Collagen")** | 24,165 |
